# TAp73 regulates ATP7A: possible implications for ageing-related diseases

**DOI:** 10.18632/aging.101669

**Published:** 2018-12-08

**Authors:** Piervito Lopriore, Nazzareno Capitanio, Emanuele Panatta, Nicola Di Daniele, Alessandra Gambacurta, Gerry Melino, Ivano Amelio

**Affiliations:** 1 MRC Toxicology Unit, University of Cambridge, LeicesterLE1 7HB, United Kingdom; 2 Department of Clinical & Experimental Medicine, University of Foggia, Foggia, Italy; 3 Department of Systems Medicine, Nephrology and Hypertension Unit, Tor Vergata University Hospital, Rome, Italy; 4 Department of Experimental Medicine and Surgery, University of Rome Tor Vergata, Rome, Italy

**Keywords:** neurodegeneration, ageing, p53 family, copper, cancer

## Abstract

The p53 family member p73 controls a wide range of cellular function. Deletion of p73 in mice results in increased tumorigenesis, infertility, neurological defects and altered immune system. Despite the extensive effort directed to define the molecular underlying mechanism of p73 function a clear definition of its transcriptional signature and the extent of overlap with the other p53 family members is still missing. Here we describe a novel TAp73 target, ATP7A a member of a large family of P-type ATPases implicated in human neurogenerative conditions and cancer chemoresistance. Modulation of TAp73 expression influences basal expression level of ATP7A in different cellular models and chromatin immunoprecipitation confirmed a physical direct binding of TAp73 on ATP7A genomic regions. Bioinformatic analysis of expression profile datasets of human lung cancer patients suggests a possible implication of TAp73/ATP7A axis in human cancer. These data provide a novel TAp73-dependent target which might have implications in ageing-related diseases such as cancer and neurodegeneration.

## Introduction

p53 family is one of the most powerful families of genes due to the large spectrum of role that plays from tumour suppression, maintenance of the cellular homeostasis, contribution to development, reproduction and ageing [1–11]. As transcription factors, the three members, p53, p63 and p73, sharing high degree of structural homology, especially in their DNA-binding domains [12], regulate the expression of genes crucial for a wide range of cellular processes, including cell cycle arrest/apoptosis, senescence, metabolism [13–19], autophagy as well as terminal differentiation in specific cell types, such as neurons for p73 [20–22] and keratinocytes for p63 [23–27]. The functional and physical interplay within the family members is also thought to play biological roles and the interaction with the mutated forms of p53 can have implications in cancer [28–36].

Similar to 95% of human genes [37,38] *TP73* gene generates multiple protein isoforms, which arise as a result of alternative promoter (P1 and P2) control and differential mRNA splicing at the 3'end. P2 activity generates ΔNp73 isoforms that lack the transactivation (TA) domain present in the N terminus of the full length p73 protein (TAp73); alternative splicing, instead, leads to 7 isoforms (α- η) varying in activity and specificity [39–41].

p73 plays critical functions in cellular processes such as neuronal development and differentiation [32,42–48], and metabolic control [42,49–57]. Mimicking p53 function, TAp73 controls cell cycle arrest and apoptosis as well as genome integrity protection in germline and somatic cells, impacting fertility and cancer [9,29,58–61]. Differently from *TP53* gene, in cancer cells p73 is rarely mutated, but shows often dysregulated expressions. Isoform-specific knockout mice revealed that the two major N-terminal p73 isoforms, TAp73 and ΔNp73, play opposite role in cancer [6,62]. TAp73 deficiency predisposes to spontaneous cancer and increases the susceptibility to carcinogens [62], whereas the absence of ΔNp73 decreases tumour growth [63]. The impact of p73 deregulation on cancer cell biology can indeed depend on the relative expression of TAp73 and ΔNp73 isoforms [64]. As a result, most studies in cancer-related fields focus their attention on the analysis of changes in TAp73 and ΔNp73 expression levels. ΔNp73 is thought to inhibit the activity of the transcriptional competent isoform TAp73, with a fine molecular tuning. ΔNp73 can indeed counteract TAp73 tetramerization or compete for promoter binding. The TAp73/ΔNp73 ratio in cells subjected to chemotherapeutic agents could therefore be crucial. TAp73 contributes to genomic stability of somatic and germline cells by controlling the mitotic checkpoints. Furthermore, TAp73 interacts with kinetochore proteins Bub1 and Bub3 to control the spindle assembly. Deregulation of TAp73 in cancer are consequentially expected to impact on polyploidy [6]. More recent work showed that TAp73 can physically bind and control stability of the hypoxia-inducible factor 1α (HIF-1α). In hypoxic tumour, expression of TAp73 represses activation of HIF-1, thus limiting tumour angiogenesis and therefore progression towards advanced stages [60,65–67]. Additional contribution of p73 to tumour cell biology might be mediated by its support to cellular anti-oxidant defence and anabolic processes. This is partially mediated by a TAp73-dependent regulation on mitochondrial proteins synthesis under oxidative stress [14] and by a transcriptional control of metabolic enzymes responsible for GSH synthesis, such as glutaminase-2 (GLS-2) [50,52] and glucose-6-phosphate-dehydrogenase (G6PD) [53].

However, despite the important effort placed by scientific community, we are very far from a dissection of p73 transcriptional programme and a clear discrimination of this from the transcriptome of the siblings p53 and p63. By re-analysing previously published high throughput genomic screening approach (gene microarrays) and filtering data by using bioinformatic tools we aimed to identify novel p73 transcriptional targets. Our analysis, supported by *in vitro* data and clinical analysis identified a previously unknown relationship between TAp73 and ATP7A (or *MNK*), a gene encoding for a transmembrane P-type ATPase transporter required for copper homeostasis in mammals [68]. ATP7A is recognized as a critical copper-transport protein with multiple important cellular functions. Mutations is ATP7A are responsible for Menkes disease, a X-linked recessive disorder characterized by growth retardation, neurodegeneration, and peculiar hair [69]. In addition, over-expression of ATP7A is observed in multiple cancers, and recent studies suggest that this copper efflux transporter play an important role in platinum drug resistance [70–76]. Our data demonstrate a TAp73-dependent *ATP7A* transcription control and a possible clinical relevance of this axis for lung cancer patients. Our finding might delineate the possible underlining mechanisms of different ageing-related conditions, such as cancer and neurodegeneration, where alterations of the TAp73/ATP7A axis might play a direct impact.

## RESULTS

### ATP7A is within the top TAp73 candidate target genes

**Figure 1:**
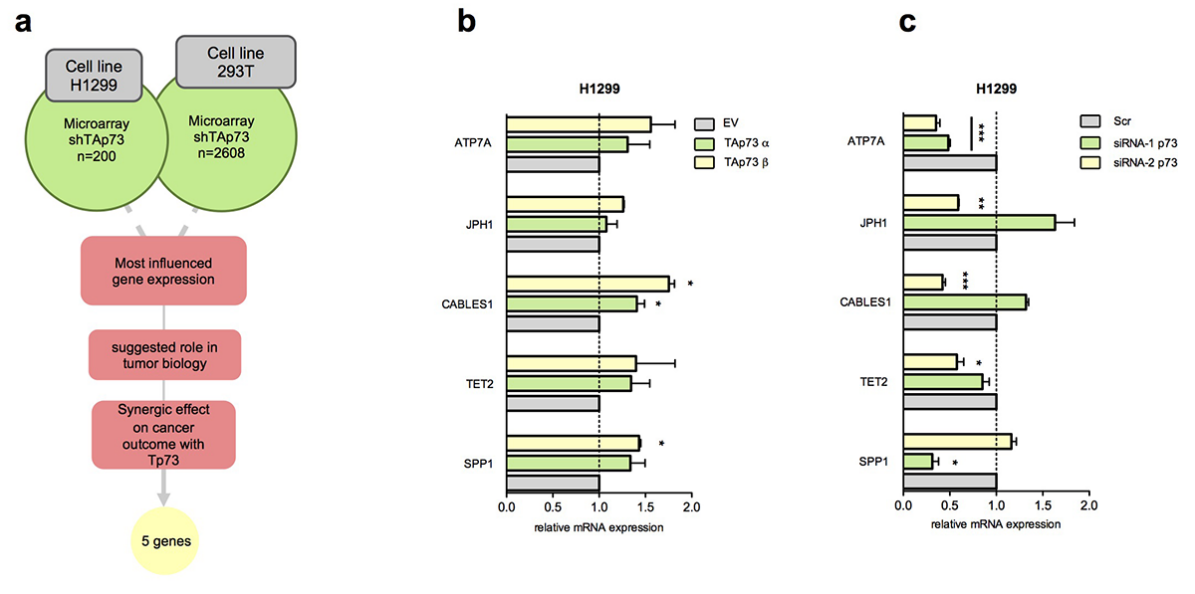
**Identification of new TAp73 transcriptional targets.** (**a**) Schematic workflow of putative TAp73 transcriptional target analysis; most influenced genes in H1299 (Amelio et al.) [65] and 293T (Marini et al.) [14] (shTAp73) were analysed by filtering ones more related with tumor biology; searching for previous evidence were done on PubMed (NCBI). Gene expressions effect on oncological patients survival and synergical effect with Tp73 on cancer outcome were analysed studying KM curves in all available datasets in web based online tool Syntarget. (**b**) mRNA levels of SPP1, TET2, CABLES1, JPH1, ATP7A were analysed by quantitative PCR after HA-TAp73 α and β overexpression. Relative expression of genes was normalized against TBP and calculated as fold change to the control treatment (empty vector, EV). Data is reported as mean ± s.d. of three experiments. * p < 0.05 (Student's T-test). (**c**) mRNA levels of genes of interest were analysed by quantitative PCR after siRNA-1 p73 and siRNA-2 p73 treatment. Relative expression of genes was normalized against TBP and calculated as fold change to the control treatment (siCTRL, Scr). Data is reported as mean ± s.d. of three experiments. *** p < 0.0001, ** p < 0.001, * p < 0.05 (Student's T-test).

To further deepen TAp73 functions as transcriptional factor we firstly started analysing two previously reported global gene expression analysis in TAp73 silenced cells, *p53*-null human non-small cell lung carcinoma cell line H1299 [65] and human embryonic kidney cell line 293T [14]. Crossing the two lists and considering the top ranked genes, in terms of fold change, we selected the ones with established role or suspected involvement in tumour biology. Hence, we tested the synergist effect of the expression of each individual gene (from a list including 175 genes) with p73 expression on survival outcome in different cancer datasets by a bioinformatic datamining tool we previously established, Syntarget [77]. Not surprisingly the most represented cancer type was lung cancer: 20 of the 21 genes found clinically synergize with *TP73* (p<0.05) in Lung Adenocarcinoma stage I-II (GSE31210) and Lung Cancer (GSE30219) datasets. Indeed, previous studies have showed that TAp73^−/−^ mice spontaneously develop lung carcinomas, and altered ratio TAp73/ΔNp73 is frequently reported in human lung cancer [62,78]. Thus, we selected 5 genes of these (*SPP1*, *TET2*, *CABLES1*, *JPH1* and *ATP7A*) that more significantly synergize with *TP73*, influencing more robustly oncological patients overall survival. A representative work flow of the followed rationale is shown in Figure 1a.

### TAp73 silencing influences ATP7A expression level

**Figure 2:**
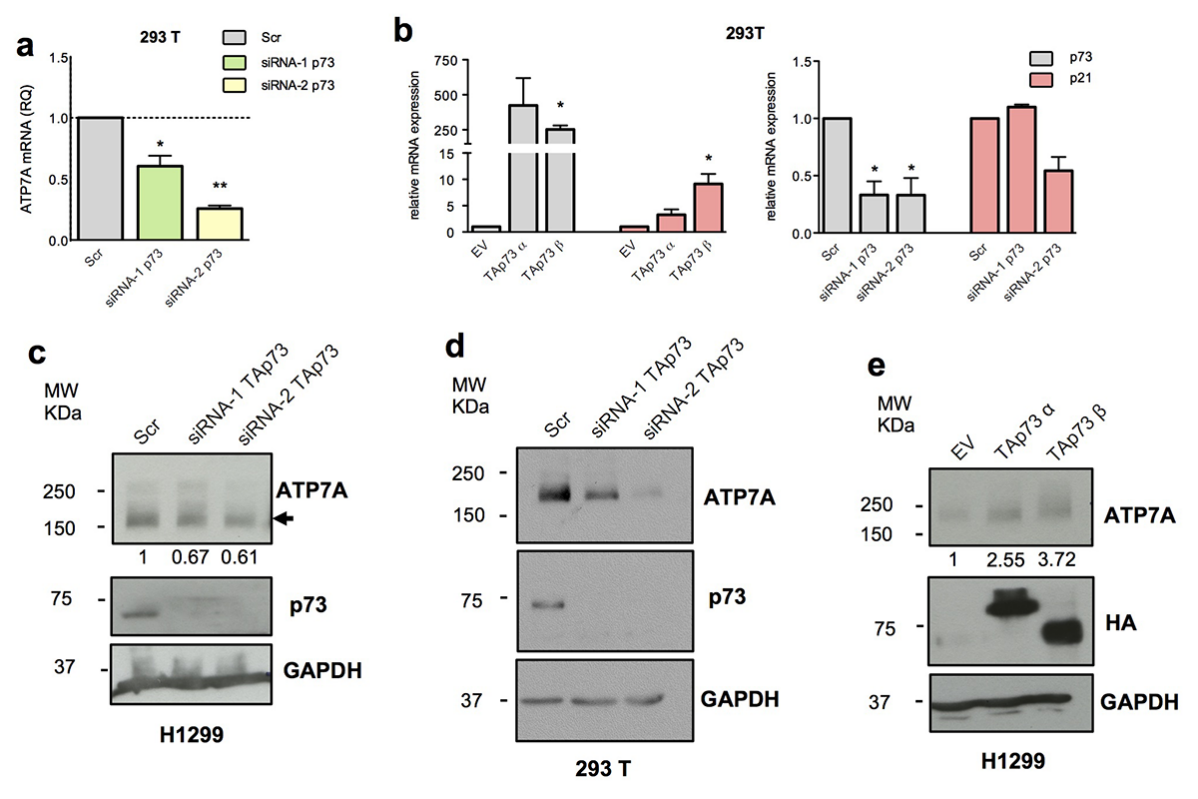
**TAp73 regulates ATP7A expression level.** (**a**) ATP7A mRNA levels were analysed by quantitative PCR after TAp73 silencing (siRNA-1/2 p73). Relative mRNA expression was normalized against TBP and calculated as fold change to the control treatment (Scr). Data is reported as mean ± s.d. of three experiments. ** p < 0.001, * p < 0.05 (Student's T-test). (**b**) p73 and p21 mRNA levels were analysed by quantitative PCR after TAp73 overexpression (HA-TAp73 α-β) and p73 silencing (siRNA-1/2 p73). Up- and downregulation of p21, a TAp73 transcriptional target, confirmed p73 transcriptional activity modulation. Relative expression of genes was normalized against TBP and calculated as fold change to the control treatments (EV and Scr). Data is reported as mean ± s.d. of two experiments. * p < 0.05 (Student's T-test). (**c-e**) Protein levels of ATP7A, HA-TAp73 or endogenous TAp73 and GAPDH were analysed by WB in cell overexpressing or depleted for TAp73. Figure shows a representative replicate of three independent experiments.

To explore potential downstream targets of TAp73 we analysed the expression level of the top 5 candidate genes following overexpression and silencing of TAp73 by transfection of pcDNA HA-TAp73 α-β and siRNA-1/2 p73 in both H1299 and 293T cell lines. p73 silencing in both cell lines resulted in a significant ATP7A transcript downregulation (Figure 1c, Figure 2a). In H1299 overexpression assay only *CABLES1*, a gene encoding for a protein well-known involved in the cell cycle regulation [79], showed slight mRNA level increase (1.5-2-fold increase) (Figure 1b). This data, however, was not confirmed in 293T cells overexpressing TAp73 (Supplementary Figure 1c). The remaining genes showed similar mRNA level fluctuation, however not always high consistency was observed in both models (Figure 1b, c and Supplementary Figure 1a, b). p73 and p21 expression levels were measured by RT-qPCR to verify efficiency of TAp73 overexpression and silencing (Figure 2b and Supplementary Figure 1c).

We decided to focus on ATP7A due to the more significant and consistent regulation observed. ATP7A mRNA reduction in p73 knocked-down cells was accompanied by a significant decrease of the protein level, more evident in the 293T model (Figure 2c,d). In addition, H1299 showed also a moderated, but consistent upregulation of ATP7A protein upon TAp73 overexpression (Figure 2e).

Collectively, these data demonstrate a TAp73-dependent regulation of *ATP7A* expression at both protein and mRNA level.

### TAp73 binds ATP7A genomic regions

The above results suggested a relationship between TAp73 and ATP7A transcript. Hence, we asked whether a TAp73-dependent transcriptional regulation of ATP7A was associated to a direct physical binding of TAp73 to ATP7A promoter region.

**Figure 3:**
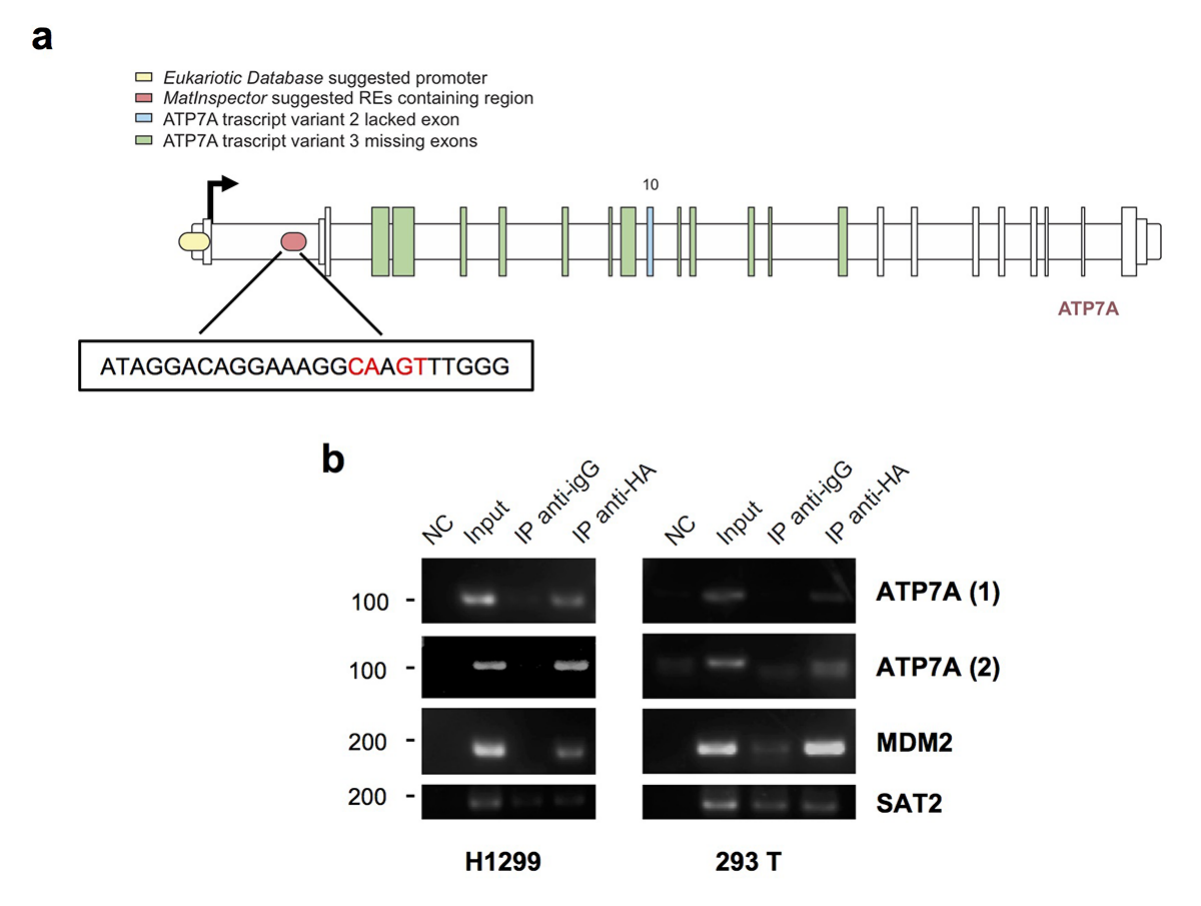
**TAp73 physically binds *ATP7A* genomic areas.** (**a**) Schematic map of the human ATP7A gene. MatInspector professional software suggested promoter region containing p53 family members-response elements is indicated in red; the insert shows the sequence of the p53 family member-RE chosen, the nearest to the TSS (ChrX: 77,961,553-77,962,65). In yellow ATP7A promoter region found on *Eukaryotic Promoter Database* by selecting a region from -1000 to 100 bp relative to TSS CAGEseq derived (ChrX: 77,909,608- 77,910,867) [Human Dec. 2013 (GRCh38/hg38)]. (**b**) A tagged TAp73 was overexpressed in H1299 and 293T cell lines for 24h. The sonicated chromatin was incubated with anti-HA or IgG antibodies. Immunoprecipitated DNA was amplified by PCR with ATP7A primers, one (ATP7A1) amplifying Eukaryotic Database suggested promoter, the other (ATP7A2) recognizing the p53-response element found on MatInspector. ChIP on MDM2 promoter was performed as a positive control, and ChIP on SAT2 promoter as a negative control. NC: PCR negative control. Figure shows a representative replicate of two independent experiments.

In addition to the canonical upstream promoter, found by using Eukaryotic Promoter Database (labelled yellow in Figure 3a), MatInspector Professional Software *in silico* analysis identified 2 regions containing responsive elements (REs) for p53-family proteins. One of these two (labelled in red in Figure 3a) included a 25 nucleotides sequence (ChrX: 77,961,553-77,962,65 – intron 1/21) with a 0.947 value of matrix similarity. Due to lower matrix similarity, we decided not to consider the other one, containing potentially 6 REs, four of them specific for p63 protein.

Immunoprecipitated chromatin with anti-HA antibody from HA-TAp73 overexpressing H1299 and 293 T cells showed specific binding of TAp73 on the REs identified in the promoter region (Eukaryotic Promoter Database) and in the intron 1 (MatInspector Professional Software) (Figure 3b). MDM2 was tested as positive control of TAp73 binding, whereas SAT2 represented a negative control.

Overall our data demonstrate a relationship between TAp73 and ATP7A. TAp73 directly binds *ATP7A* promoter region and the RE localized in intron 1, suggesting a potential direct transcriptional control, and depletion of TAp73 impact on the basal expression level of ATP7A. However, TAp73 overexpression appears not sufficient to strongly promote *ATP7A* expression level.

### p73/ATP7A axis in human cancer

In order to better understand the clinical relevance of p73-dependent regulation of ATP7A in human cancer, we evaluated by computational webtools the impact of the correlation between p73 and ATP7A in different publicly available datasets of expression profiling of oncologic patients.

**Figure 4:**
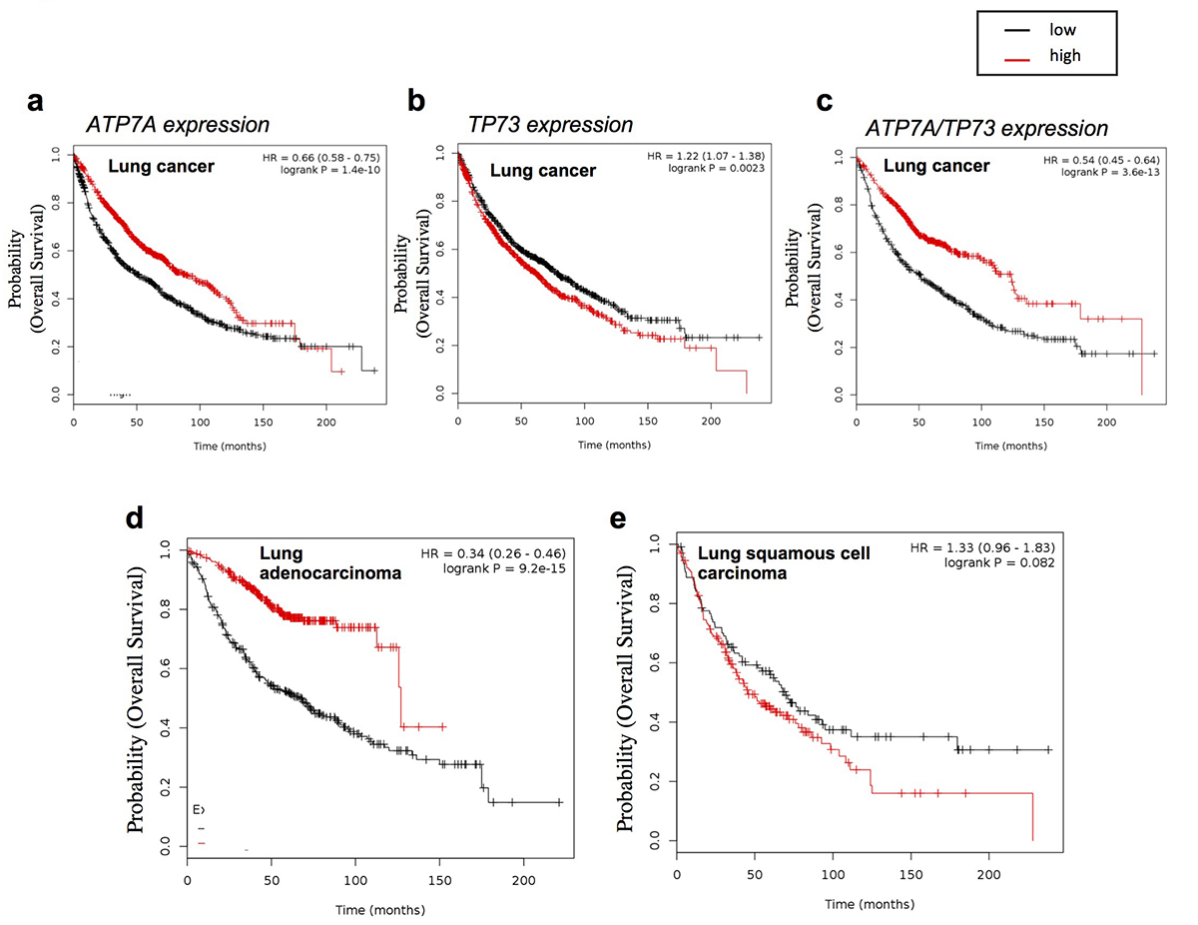
**ATP7A expression affect lung cancer patient’s outcome.** Overall survival analysis of lung cancer datasets (see Material and Methods for full list) relative to (**a**) Tp73 expression (232546_at probe, mean expression) (n=1926), (**b**) ATP7A expression (205198_s_at probe, mean expression) (n=1145), (**c**) Tp73 and ATP7A probes mean expression (n=1145). HGU133A probe set. Patients splitted by median. P values showed on the top. Overall survival analysis of lung cancer datasets relative to ATP7A expression (205198_s_at probe, mean expression) obtained filtering the results with (**d**) ”Adenocarcinoma” (n=673) and (**e**) “squamous cell carcinoma” histology type (n=271). HGU133A probe set. Patients split by median. P values showed on the top.

Using human lung cancer datasets we assessed the impact of the expressions of the individual genes and the combination of both on the patient survival. Unexpectedly ATP7A high expression positively impacted on patient's survival (*P* value 1.4e-10) (Figure 4a), whereas high p73 expression had a marginal but negative impact (*P* value 0.0023) (Figure 4b). However interestingly the combination of high expression of both genes together highly significantly influenced the overall survival of lung cancer patients (*P* value 3.6e-13) (Figure 4c).

An important major difference was observed when the lung cancer datasets were selected for the different histological subtype. ATP7A expression was importantly discriminating good and bad prognosis in lung adenocarcinoma patients (*P* value 9.2e-15), but no effect was observed in the subset of samples belonging to the squamous cell carcinoma histological subtype (Figure 4d, e).

Despite a potential unexpected result, this analysis indicated a possible implication of p73/ATP7A axis in human lung cancer. According to previously observed role of ATP7A in chemoresistance, a high expression level would have been expected to define a poor prognosis cohort, however unexpectedly our result indicates that high level of ATP7A positively influences patient's prognosis. In addition, high expression of both ATP7A and p73 is defining a subgroup with a significant better prognosis compared to the individual contribution of ATP7A and p73. This result indicates that the activation of p73/ATP7a axis could play a tumour suppressor role in a subgroup of lung cancer patients.

## DISCUSSION

20 years after the discovery of the p53 homologue, p73, we still lack a clear definition of its transcriptional signature and the extent of overlap with the ones controlled by the other p53 family members [80–82]. Here we reanalyse previously published transcriptional analysis of TAp73 depleted cells, trying to investigate potential novel transcriptional targets responsible for TAp73 function.

We describe a novel TAp73 target, ATP7A, that in our cellular models appears to require TAp73 expression in order to maintain its basal expression level. However, following TAp73 overexpression despite we observe TAp73 accumulation on ATP7A genomic region, no strong effects are observed on ATP7a transcript and protein level. This result might have multiple explanations. For a certain extent this highlights the complexity of specific transcriptional networks. TAp73-dependent ATP7A regulation could indeed not simply rely on the direct TAp73 binding on the ATP7A genomic sites, but require the interplay with additional transcriptional factors. Previous studies in zebrafish models have showed that *SOD1* transcription is affected by ATP7A in a copper responsive manner through the transcription factor Sp1 [83–85]. Moreover, Sp1 itself binds *ATP7A* promoter in human intestinal epithelial cells being fundamental for the HIF2α-mediated induction of gene transcription during iron deficiency/hypoxia to regulate iron balance [86]. Interplay between TAp73 and HIF family proteins [65], Sp1 transcription factor [87,88] or a hierarchical genetic regulation mediated by copper could be therefore involved in this regulatory mechanism. Downstream pathways altered by TAp73 need to be elucidated in the specific context of ATP7A regulation to fully clarify all the participants of this signalling. An alternative explanation of our results could be that TAp73 has the capacity of controlling alternative transcriptional signatures in a dosage-dependent manner. High expression level of TAp73 could be responsible for upregulation of pro-apoptotic/cell cycle arrest genes in contexts such as genotoxic stress. Basal expression level of TAp73 instead might be responsible for the control of homeostatic and/or metabolic regulators. These two transcriptional programmes might be completely distinguished and might be determined by the expression level of TAp73. This would represent a potential similarity with p53 functions in DNA damage response and basal conditions [89–93].

The biological significance of our findings is strictly associated to the lack of clarity regarding the implications of p73 for human diseases. KO mouse models for p73 show a range of defects that include tumour suppression, infertility, neurological defects and altered immune system [47,94]. The complexity of p73 functions is therefore obviously highlighted by the phenotype of the genetic mouse models. The control of genomic stability is central to all family members, and TAp73 deficiency is associated with genomic instability that emerged to be important not only in tumorigenesis but also in maturation of oocytes. In addition to this, more recently a defective ciliogenesis of multiciliated epithelia, such as the upper airway tract, further expanded the range of defects observed in p73 mouse models. However, despite that, a clear connection of p73 with human disease never emerged. p73 deficiency in mice is also associated to premature ageing.

The altered mitochondrial metabolism is a root cause for premature ageing and mitochondrial dysfunction in TAp73 KO mice plays a key role in this context [95–101]. Oxidative damage promotes cellular senescence *in vitro* and ageing *in vivo*. TAp73-null MEFs and silenced cells are sensitive to oxidative damaging agents such as hydrogen peroxide (H2O2). On the other hand, TAp73-null MEFs grow well in low-oxygen conditions or with the addition of antioxidants, conditions that dampen oxidative damage [54,102,103]. These indicates that an important part of p73 functions depends on its control of cellular metabolism.

ATP7A is a member of a large family of P-type ATPases, which are energy-utilizing membrane proteins that pumps ions and lipids across cellular membranes [104]. ATP7A has a dual homeostatic and biosynthetic functions: exporting copper in excess outside the cell, and transporting copper to cuproenzymes at the secretory pathway [105]. Depending on the copper intracellular concentration and the cellular states, ATP7A can be shuttled from the endoplasmic reticulum to the plasma membrane, facilitating copper extrusion from the cell. Mutations in ATP7A leads to Menkes Disease, a lethal paediatric multisystemic disorder associated to progressive neurodegeneration [106].

The TAp73/ATP7A axis might play a role in different biological contexts, with a potential high interest in ageing-associated diseases, such as cancer and neurodegeneration. Our computational analysis of expression profiling datasets of human cancer highlighted a potential correlation of TAp73/ATP7A with cancer pathogenesis; further studies will determine if this is a simple correlative connection, or an actual causative relationship. Despite a role in human cancer of ATP7A has been identified in drug-resistance (drug extrusion from cancer cells), our data indicate that high level of ATP7A is a positive prognostic factor, which becomes even stronger when concurrent with high TAp73 expression. The data are therefore suggestive of a potential implication of TAp73/ATP7A axis in tumour suppression.

Implication of TAp73 in mouse neurodevelopment and deregulation of TAp73 expression in human neurodegenerative conditions might be suggestive of role of TAp73/ATP7A axis also in neuro-biology. Possibly the altered TAp73-dependent regulation of ATP7A during ageing could produce neuro-toxic effects responsible for progressive neurodegeneration similar to the severe manifestations observed in Menkes Disease. However, in absence of any experimental evidence these speculations might only indicate potential future research directions aimed to better define p73 implications in human diseases.

## MATERIALS AND METHODS

### Cell Culture

The human non-small cell lung carcinoma cell line NCI-H1299 were cultivated in RPMI medium 1640 (Gibco, Life Technologies, Carlsbad, CA, USA) containing 4.5 g/L D-Glucose, 2.383 g/L HEPES Buffer, L-Glutamine, 1.5 g/L Sodium Bicarbonate, and 110 mg/L Sodium Pyruvate, supplemented with 50 units/mL Penicillin, 50 mg/mL Streptomycin (Gibco), and 10% (vol/vol) FBS (Labtech, Heathfield, UK). The human embryonic kidney cell line HEK-293 were cultivated in Dulbecco’s Modified Eagle Medium (Gibco) containing 4.5 g/L D-Glucose, L-Glutamine, and Pyruvate, supplemented with 50 units/ mL Penicillin, 50 mg/mL Streptomycin (Gibco), and 10% (v/v) FBS (Labtech). All cell cultures were maintained 37 °C with 5% CO2 in a humidified incubator.

### Cell transfection

For overexpression, H1299 cells were seeded 24 h before transfection. Transfection was performed with 10 μg DNA (pcDNA empty, pcDNA HA-TAp73α, pcDNA HA-TAp73β) per 10 cm dish with 1.2 × 10^6^ cells seeded using Lipofectamine 2000 Reagent (Invitrogen). Cells were collected 24 h after transfection. For 293T cells transfection was performed with 3 μg DNA per 10 cm dish with 2.5 × 10^6^ cells seeded using Effectene reagent (Qiagen, Manchester, UK) and cells collected 24 h after transfection. For p73 knockdown in H1299 and 293 T 1.2 × 10^6^ and 2.5 × 10^6^ cells, respectively, were seeded per 10 cm dish 24 h before transfection. Transfection was performed using 50 nM siRNA [control siRNA, siRNA-2 p73-1 and siRNA-2 p73-2 (Ambion)] and Lipofectamine RNAiMAX (Invitrogen). Cells were collected 48 h after transfection.

### RNA extraction and analysis

Total RNA was isolated from cells using the RNEasy Mini Kit (Qiagen), according to the Qiagen company protocol. 2 μg of total RNA was used to prepare cDNA using RevertAid H minus First strand cDNA Synthesis kit (ThermoScientific), using Random primers and the protocol from the kit. qPCR was performed using 1/10 of the prepared cDNA and Power SYBR Green PCR Master Mix (Applied Biosystems). Relative gene expression was analyzed in accordance with 7500 Software version 2.0.6 of Applied Biosystems. Gene expression levels were quantified according to the comparative ΔΔCt method and normalized to expression of the TBP housekeeping gene. Sequences of the primers used for the qPCR are:

TAp73 Fw CAGACAGCACCTACTTCGACCTT, Rev CCGCCCACCACCTCATTA;  p21 Fw CCTGTCACTGTCTTGTACCCT, Rev GCGTTTGGAGTGGTAGAAATCT; TBP Fw TCAAACCCAGAATTGTTCTCCTTAT, Rev CCTGAATCCCTTTAGAATAGGGTAGA; SPP1 Fw GAGGGCTTGGTTGTCAGC, Rev CAATTCTCATGGTAGTGAGTTTTCC; CABLES1(transcript variant 2) Fw TCGCGACAGTACCCAAGTC, Rev TCAAACTCACTGCACCAGTTG; TET2 Fw AAAGATGAAGGTCCTTTTTATACCC, Rev ATAGCTTTACCCTTCTGTCCAAAC; JPH1 Fw GACATCGCGAGAGCTGTG, Rev TTCCTGAAATCTCTGTTTGACG; ATP7A (transcript variant 1) Fw TCTTCCAGGATTGTCTGTTATGAA, Rev ACCAGCTCCGAAAAACTG;  ATP7A (transcript variant 2) Fw TCTTCCAGGATTGTCTGTTATGAA, Rev CCTCTGATGTTTTGCCCTGTA;

ATP7A (transcript variant 3) Fw TGTGCATCACATTAAGGTAAAGGTA, Rev AGTTCCCACAATGGCCAAGA.

### Western blotting

For protein extraction, cells were lysed in RIPA buffer with protease inhibitor cocktail tablets Complete, EDTA-free (Roche) and phosphatase inhibitor cocktail tablets PhosSTOP (Roche). Lysates were measured for protein concentration by using the Bio-Rad Protein Assay (Bio-Rad), then mixed with Laemmli loading buffer, electrophoresed on SDS-PAGE gels and separated proteins transferred to PVDF blotting membranes (Amersham, GE Healthcare). Membranes were blocked for 1 h in 10% (m/vol) dry milk dissolved in TBS with 1% (vol/vol) Tween-20 (TBSt); incubated with primary antibodies overnight and with secondary antibodies conjugated with horseradish peroxidase, for 1 h. Antibodies were diluted in 10% dry milk in TBSt: anti-HA 1:1000 (Covance), anti-GAPDH 1:40000 (Sigma), anti-p21, anti-p73 1:3000 (Bethyl), anti-ATP7A 1:500 (Santa Cruz). To detect the signal ECL Western Blotting Detection Reagent (Amersham, GE Healthcare) or SuperSignal West Dura Chemiluminescent Substrate (Thermo Scientific) was used.

### Promoter region analysis

Analysis of promoter region was performed using MatInspector Professional software by Genomatix (https://www.genomatix.de) and the Eukariotic Promoter Database (https://epd.vital-it.ch/index.php).

### Chromatin Immunoprecipitation assay

TAp73α was overexpressed for 24 h in H1299 and 293T cell lines (see cell transfection section for details). Cells were collected fixed in 37% formaldehyde and subjected to sonication for DNA shearing. Chromatin was sonicated (around 500 bp) and immunoprecipitated with/without 10 μL anti-HA antibodies (Covance) or 10 μL nonspecific immunoglobulin G (IgG) antibodies (Invitrogen) using the MAGnify ChIP System kit (Invitrogen). The co-immunoprecipitated DNA fragments were amplified by PCR. MDM2 was used as positive control. SAT2 was used as negative control.

ATP7A (1) Fw GGTTTCGCTTTTGTCGTGGG, Rev TGAAAAGGAACGCGTGGTCT; ATP7A (2) Fw ATACCCTTGTACTGCTTCCCAC, Rev TAGGATGAGTTCAGGTGGCG; MDM2 Fw GGTTGACTCAGCTTTTCCTCTTG, Rev GGAAAATGCATGGTTTAAATAGCC;  SAT2 Fw CTGCAATCATCCAATGGTCG, Rev GATTCCATTCGGGTCCATTC.

### Bioinformatic analyses

First bioinformatic analysis, to test gene synergy in cancer, was performed using *Syntarget* by ChemoProfiling (http://www.chemoprofiling.org). All available datasets had been checked. Survival analysis on lung cancer patients was performed by Kaplan-Meier Plotter (http://kmplot.com/analysis/). Datasets considered were: CAARRAY, GSE14814, GSE19188, GSE29013, GSE30219, GSE31210, GSE3141, GSE31908, GSE37745, GSE43580, GSE4573, GSE50081, GSE8894, TGCA.

### Statistics

Technical as well as biological triplicates of each experiment were performed. Error bars indicate ±S.D. in each figure. Statistical significance was determined using the unpaired two-tailed Student's *t*-test using GraphPad Software. A p-value ≤0.05 was considered statistically significant.

## SUPPLEMENTARY MATERIAL

Supplementary Figure 1
