# Supplementary material for: TAp73 regulates ATP7A: possible implications for ageing-related diseases
Source: Aging (Albany NY). 2018 Dec 8;10(12):3745–60. doi: 10.18632/aging.101669 (PMC6326685; doi:10.18632/aging.101669)
Supplement: Supplementary Figure 1 [file aging-10-101669-s001.pdf]

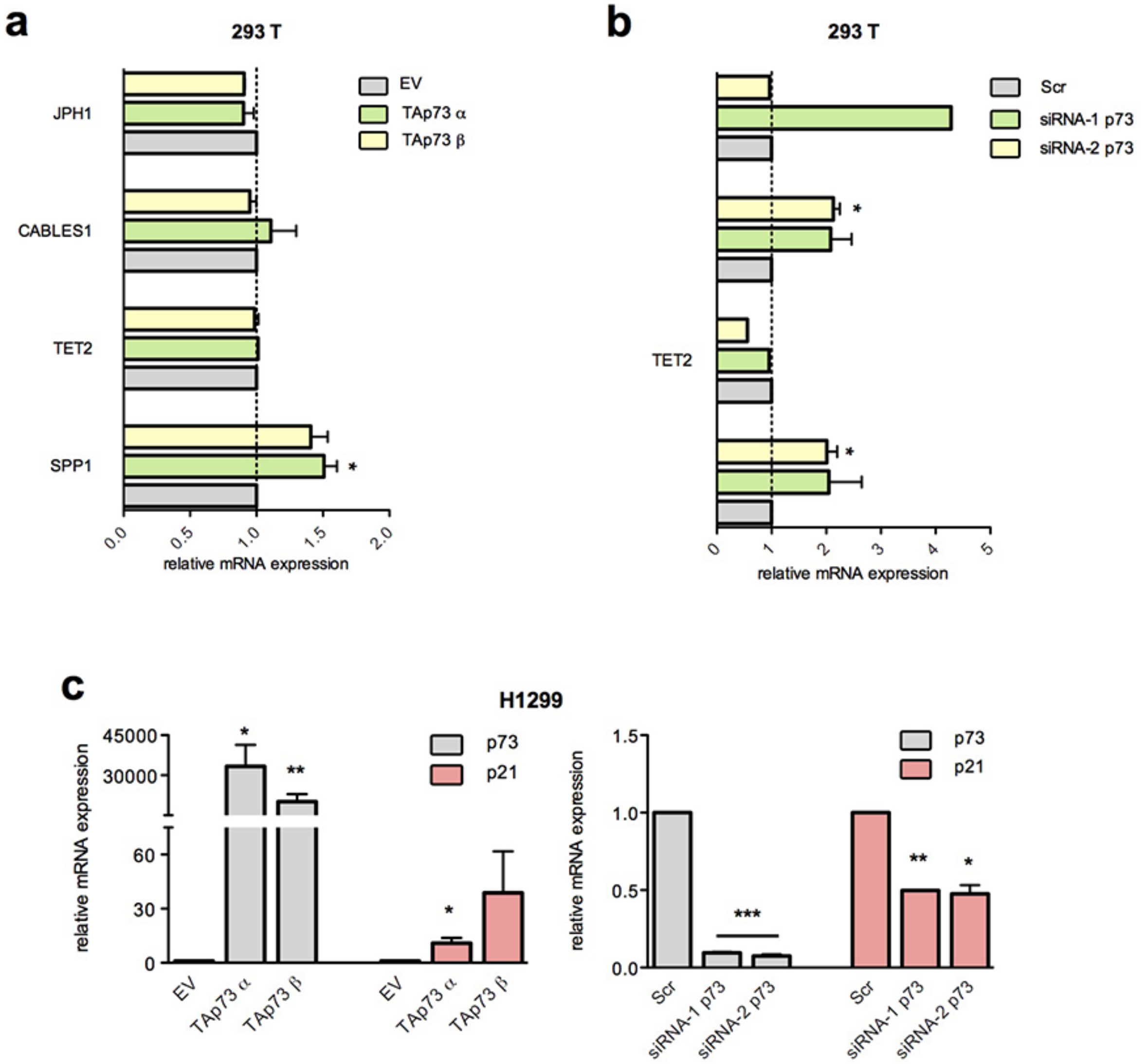

Supplementary Figure 1. TAp73 overexpression and silencing in 293T cells and H1299. (**a**) mRNA levels of SPP1, TET2, CABLES1, JPH1, ATP7A were analysed by quantitative PCR after HA-Tap73 α and β induction. Relative expression of genes was normalized against TBP and calculated as fold change to the control treatment (empty vector, EV). Data is reported as mean ± s.d. of two experiments. * $p < 0.05$ (Student's T-test). (**b, c**) mRNA levels of genes of interest were analysed by quantitative PCR after siRNA-1 p73 and siRNA-2 p73 treatment. Relative expression of genes was normalized against TBP and calculated as fold change to the control treatment (siCTRL, Scr) Data is reported as mean ± s.d. of two experiments. * $p < 0.05$ (Student's T-test). (**c**) p73 and p21 mRNA levels were analysed by quantitative PCR after TAp73 overexpression (HA-Tap73 α-β) and p73 silencing (siRNA-1/2 p73). Up- and downregulation of p21, a TAp73 transcriptional target, confirmed p73 transcriptional activity modulation. Relative expression of genes was normalized against TBP and calculated as fold change to the control treatments (EV and Scr). Data is reported as mean ± s.d. of three experiments. *** $p < 0.0001$, ** $p < 0.001$, * $p < 0.05$ (Student's T-test).
